# Knockdown of long non-coding RNA NEAT1 relieves the inflammatory response of spinal cord injury through targeting miR-211-5p/MAPK1 axis

**DOI:** 10.1080/21655979.2021.1930925

**Published:** 2021-06-21

**Authors:** Qing An, Zipeng Lu, Yi Xie, Yu Li, Haixiang Wei, Yang Cao

**Affiliations:** a Department of Medicine, Soochow university, China; b Hand Surgery Department, The First Affiliated Hospital of JinZhou Medical University, China; c Bone Trauma Department, The First Affiliated Hospital of JinZhou Medical University, China

**Keywords:** Lncrna, neat1, spinal cord injury, miR-211-5p, mapk1

## Abstract

Spinal cord injury (SCI) is a refractory disease often accompanied by inflammation. Long non-coding RNA NEAT1 (lncRNA NEAT1) was reported to be involved in the expression of the inflammasomes, while the regulatory effect of NEAT1 on SCI was poorly investigated. Herein, we carried out further studies on the pathogenesis of SCI. PC-12 cells were incubated with lipopolysaccharide (LPS) to induce inflammation. Western blotting assay was used to measure the protein expression levels. RNA expression levels were analyzed using RT-qPCR. Cell counting kit 8 and flow cytometry assays were used to separately determine the cell viability and apoptosis rate. The targeted relationships were verified by luciferase reporter and RNA pull-down assays. It was found that LPS induced inflammation in the PC-12 cells, leading to significantly higher cell apoptosis rate and lower viability, and the expression level of NEAT1 was elevated by LPS. However, knockdown of NEAT1 partially reversed the effects of LPS. Subsequently, the potential interaction between NEAT1 and miR-211-5p was validated and miR-211-5p inhibitor was further confirmed to antagonize the effects of NEAT knockdown. The downstream target gene of miR-211-5p was predicted and verified to be MAPK1. In addition, overexpression of MAPK1 was proved to antagonize the effects of NEAT1 knockdown. Taken together, the knockdown of NEAT1 remarkably alleviated the inflammation of SCI via miR-211-5p/MAPK1 axis.

## Graphical abstract

**Figure:**
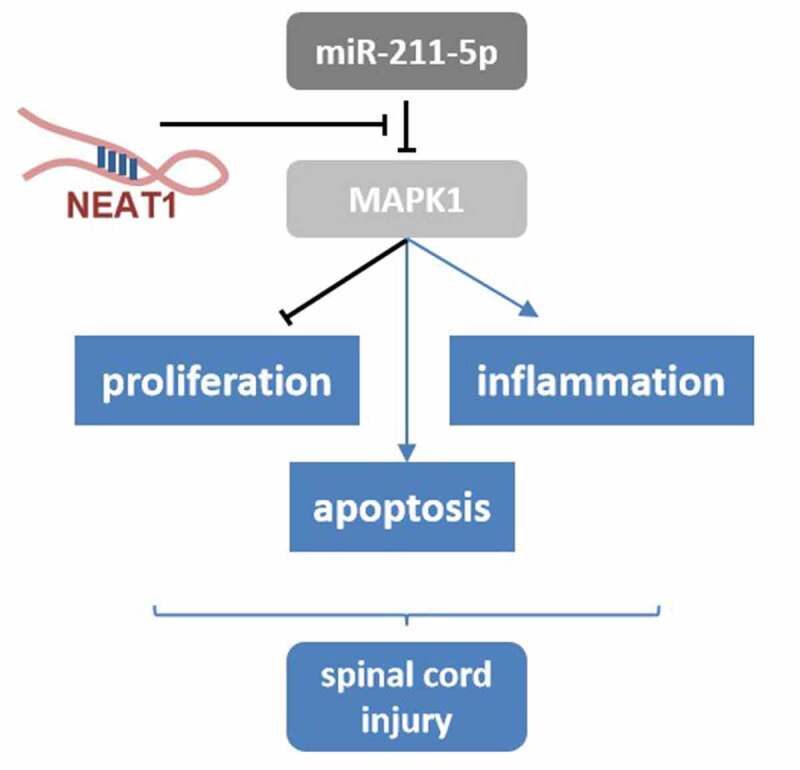

## Introduction

Spinal cord injury (SCI) is a highly morbid neurological dysfunction, and its most typical manifestation is paralysis below the plane of injury [1]. The occurrence of SCI often ascribes to damages on the intraspinal nerve system (including spinal cord and nerve roots) caused by diversified pathogenic factors (trauma, inflammation, tumor), which result in a high disability rate [2]. SCI can be divided into two categories: traumatic and non-traumatic types according to pathogenic factors. The proportion of traumatic SCI is 85.66% [3]. Statistics showed that SCI incidence was 12.7–29.7 per million in developing countries, while the incidence in developed countries was about 13.1–52.2 per million [4]. The pathogenesis of SCI was complex, involving two stages of primary and secondary injury. After the occurrence of primary injury such as fractures, a series of secondary injuries (inflammation, tissue hypoxia, neuronal necrosis and apoptosis) further aggravated SCI, leading to severe sensory and motor functions loss [5]. Despite timely medical treatments, a considerable number of SCI patients still suffered from paralysis throughout their lives [6], which brought heavy burdens to individuals and society. Therefore, it is of extraordinary significance to find new molecular targets that can effectively relieve the disease.

Long-non coding RNA (lncRNA) is a subgroup of non-coding RNA transcripts with more than 200 nucleotides. It exhibits biological effects by interacting with genomic DNA, micro-RNA (miRNA), mRNA and protein. LncRNA nuclear enriched abundant transcript 1 (lncRNA NEAT1) is mainly distributed in the nucleus, and is an important structural molecule in the substructure of the nuclear paraspeckle. It is essential for maintaining the structural integrity of the paraspeckle. NEAT1 was involved in the pathological process of viral infections, neurodegenerative diseases and the occurrence and development of various tumors. It sponges microRNA-124-3p to up-regulate phosphodiesterase 4B (PDE4B) and accelerate the progression of Parkinson’s disease [7]. NEAT1 was also reported to be related to inflammation reaction, while the function of NEAT1 on SCI was poorly investigated.

In recent years, studies demonstrated that miR-211-5p had therapeutic functions on melanoma and other tumors [8–10]. Mature miRNA is assembled into the RNA-induced silencing complex (RISC), which recognizes the 3ʹUTR region of target gene mRNA by complementary base pairing, and guides the RISC to degrade the target mRNA or block the mRNA translation, thereby negatively regulating the expression of the target gene [11]. Mitogen-activated protein kinase 1(MAPK1) is an extracellular signal-regulated kinase, which comes from MAPK family. It participates in various of cell processes including proliferation, differentiation, transcription regulation and development [12,13]. The expression of MAPK1 had been found to be dysregulated in many cancers, and the specific mechanism still remained unknown [14,15].

We hypothesise that NEAT1 sponges miR-211-5p to upregulate the MAPK1 expression. The aim of this study was to investigate the regulatory relationship among NEAT1, miR-211-5p and MAPK1 in the lipopolysaccharide (LPS)-evoked SCI cell model. Our goal is to extend our understanding of the mechanisms of SCI and to find potential therapeutic targets for the treatment of spinal cord injury.

## Materials and methods

### Cell culture

PC-12 cell lines were purchased from Procell. The cells were resuspended at a density of 5 × 10^4^ cells/ml following cell thawing in DMEM (Solarbio, Beijing, China) containing streptomycin (100 U/ml), penicillin (100 U/ml) and 10% FBS (all from Gibco). The PC-12 cells were incubated at 37°C in the presence of 5% CO_2_ (Radobio) until cell confluence reached 70%-80%. Then, the cells were collected for the following experiments.

### SCI model

The cells were resuspended to reach a final concentration of 1.0–2.0 × 10^6^ cells/ml and subsequently plated into a 96-well plate at 1 ml per well. A series of gradient concentrations including 0, 2, 4, 6, 8 μg/ml of LPS (Beyotime Institute of Biotechnology) were added to induce SCI in the cell and grown at 37°C, in the presence of 5% CO_2_ for 24 h. These cells were used for further investigation.

### Cell counting kit 8 (CCK8) assay

The cells received transfection and LPS treatment were harvested and seeded into 96-well plates at the density of 1 × 10^4^ cells/well. CCK8 Kit (HY-K0301, MedChemExpress) was used to detect the cell viability. 10 μl of CCK8 reagent was added to each well of the plate and subsequently cultured in the incubator at 37°C in the presence of 5% CO_2_ for 2 h. The absorbance values were evaluated with a microplate reader (51,119,000, Thermo Fisher Inc.).

### Cell transfection

miR-211-5p mimics, inhibitors, NEAT1 small interfering RNA (si-NEAT), MAPK1 overexpression vectors and their negative controls (RiboBio, Guangzhou, China) were transfected into the cells using Lipofectamine® 2000 reagent (Invitrogen). After 6 to 8 hours of culture, change the medium to complete medium, and continue to culture for 24 to 48 hours for subsequent experiments.

### Quantitative real-time PCR (qRT-PCR)

After extracting the total RNA with Trizol reagent (Invitrogen, USA), 5 μg of RNA was reverse transcribed into cDNA according to the instructions of the reverse transcription kit (Takara, Dalina, China). qRT-PCR reaction was carried out in a FAST7500 realtime-PCR system (ABI, USA). Reaction conditions were as follows: pre-denaturation at 96°C for 5 min, denaturation at 96°C for 30 s, annealing at 54°C for 30 s, extension at 72°C for 30 s, and 30 cycles were repeated; finally, extension at 72°C was 10 min and stored at 4°C. The relative expression level was calculated using the 2^−ΔΔCt^ method. GAPDH and U6 were used as the internal control for the mRNAs and miRNAs, respectively.

### Western blotting

Cells were lysed and the total protein was separated by RIPA buffer (Beyotime Institute of Biotechnology). BCA Protein Assay Kit (ab102536; Abcam) was used to evaluate the protein concentrations. Anti-bax, anti-caspase-3, anti-cleaved caspase-3, anti-bcl-2, anti-GAPDH primary antibodies (1:1000) and horseradish peroxidase-conjugated secondary antibody (1:1000) were purchased from Abcam. Western blotting procedure was conducted as previously described [16]. 40 μg of protein was separated with the sodium dodecyl sulfate-polyacrylamide gel (SDS-PAGE) electrophoresis. The voltage is 80–100 V electrophoresis for 3 h. After electrophoresis, the protein was transferred to the Polyvinylidene fluoride (PVDF) membrane by electrotransfer method. After blocked and rinsed in phosphate buffer containing skimmed milk powder and Tween-20 at 4°C, the blots were incubated with the primary antibodies overnight at 4°C followed by incubating with the goat anti-rabbit secondary antibody at room temperature for 2 h. Finally, the protein bands were detected by the enhanced chemiluminescence reaction and further analyzed by ImageJ software (version 1.52).

### Flow cytometry apoptosis assay

After transfection and LPS treatment, the cells were resuspended at a density of 1 × 10^6^ cells/ml. Apoptotic cells were stained by Annexin V-FITC/PI Apoptosis Detection Kit (40302ES50; Yeasen Biotech Co., Ltd.) according to the instructions. Then, the cells were examined using CytoFLEX™ Flow Cytometer. Cell apoptosis was analyzed using CytExpert software (both from Beckman Coulter, Inc.).

### Dual-luciferase reporter assay

The wild- and mutant-type of NEAT1 and MAPK1 were synthesized and cloned into luciferase reporters by RiboBio. MiR-211-5p mimic or mimic control along with the luciferase reporter were co-transfected into the PC-12 cells. After 24 h incubating, the cells were lysed and tested luciferase activity followed by the Promega Dual-Luciferase® Reporter Assay System’s manual (cat no. E1960). *Renilla* was used as an internal reference. The ratio of firefly fluorescence intensity of Renilla fluorescence intensity reflects the relative fluorescence intensity of each group.

### RNA pull-down assay

The biotin-labeled miR-211-5p probes were constructed by RiboBio (Guangzhou, China). Lysate from the cells was mixed with the probes and incubated at room temperature for 2 h. Then, PuriMag® G-streptavidin magnetic beads were added to the mixture and incubated for another 4 h. Beads were rinsed six times in the lysis buffer. The retrieved RNA was detected by RT-qPCR.

### Statistical analysis

Statistical analysis was performed using GraphPad Prism (version 8.2.1.441, GraphPad Software Inc.). The results were presented as mean ± SD. The student t-test was performed for the comparison between two groups, and the analysis of variance (ANOVA) was used for the comparison among multiple groups. Correlation analysis was achieved by Pearson’s correlation coefficient. P < 0.05 stood for statistically significant difference.

## Results

In the present study, we hypothesized that NEAT1 sponges miR-211-5p to upregulate the MAPK1 expression and participates in the regulation of the spinal cord injury. The aim of this study was to investigate the regulatory relationship among NEAT1, miR-211-5p and MAPK1 in the lipopolysaccharide (LPS)-evoked SCI cell model. We established LPS induced PC-12 inflammatory injury model to investigate the functions of NEAT1 and miR-211-5p. Mechanistically, we explored the interaction between NEAT1 and miR-211-5p as well as miR-211-5p and MAPK1.

### *LPS aggravates inflammatory response and promotes NEAT1 expression* in vitro

**Figure 1.**
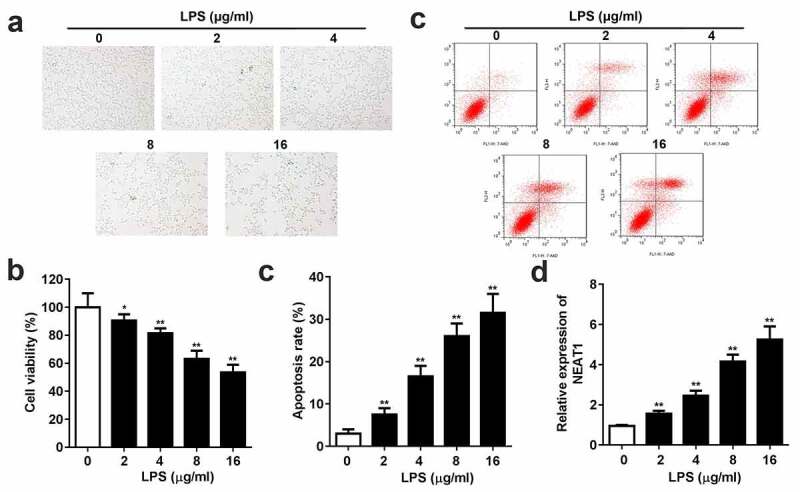
Inflammation and NEAT1 expression are promoted in the LPS-evoked cells. (a) Cell viability and (b and c) apoptosis rate of the PC-12 cell induced by 0, 2, 4, 8, 16 μg/ml LPS. (e) Expression levels of NEAT1 in the cells incubated with 0, 2, 4, 8, 16 μg/ml LPS. Three independent experiments were carried out. *P < 0.05, vs. 0 μg/ml LPS; **P < 0.01, vs. 0 μg/ml LPS. LPS, lipopolysaccharide; NEAT1, nuclear enriched abundant transcript 1

Different concentrations of LPS were used to induce SCI in PC-12 cells. CCK8 and flow cytometry assays demonstrated that LPS markedly reduced cell viability and enhanced cell apoptosis rate (Figure 1a-c). Moreover, the dose-dependent expression level of NEAT1 was exhibited in Figure 1d, LPS incubation remarkably elevated the expression level of NEAT1.

### Knockdown of NEAT1 increases cell viability and restrains cell apoptosis and inflammation

**Figure 2.**
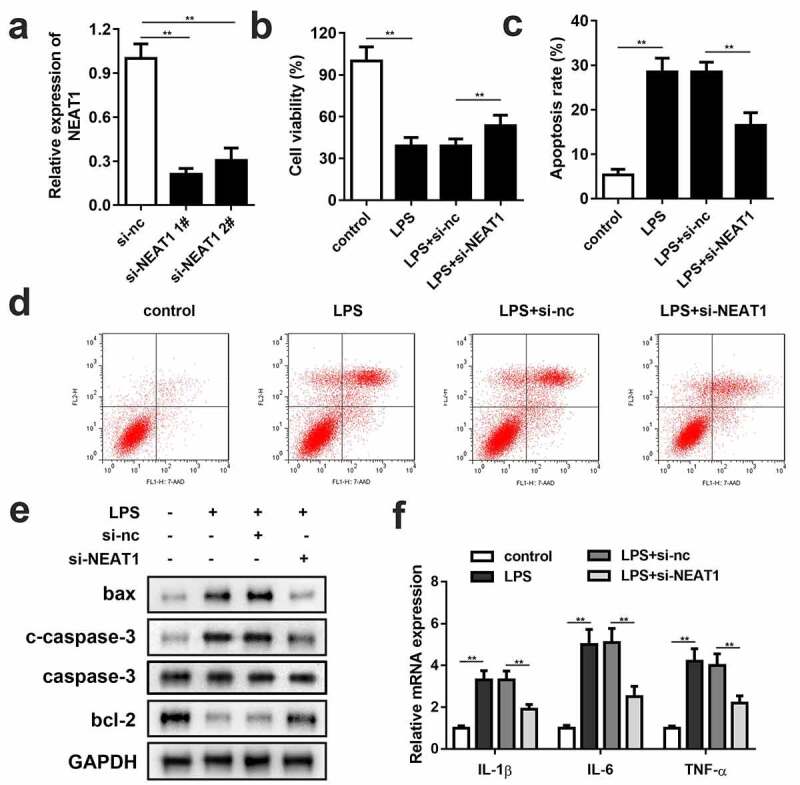
Knockdown of NEAT1 enhances cell viability, and restrains cell apoptosis and inflammation. (a) Si-NEAT1 was successfully synthesized and transfected into the cells. (b) Cell viability and (c and d) apoptosis of the control group and the LPS-evoked cells transfected with si-NEAT1. (e) The protein expressions of bax, cleaved caspase-3 and bcl-2. (f) mRNA expressions of IL-1β, IL-6, IL-10, and TNF-α in the control group and the LPS-evoked cells transfected with si-NEAT1. Three independent experiments were carried out. **P < 0.01, vs. control or LPS+si-nc. LPS, lipopolysaccharide; si, small interference RNA; NEAT1, nuclear enriched abundant transcript 1; nc, negative control

Small interference RNA of NEAT1 (si-NEAT1) 1# and 2# were successfully constructed and transfected into the PC-12 cells (Figure 2a). Since there was no significant difference between 1# and 2# on NEAT1 expression level, si-NEAT1 1# was chosen for the subsequent experiments. Knockdown of NEAT1 partially reversed the repressive effect of LPS on cell viability (Figure 2b). Besides, the cells transfected with si-NEAT1 showed effective remission on inflammation and cell apoptosis. Flow cytometry assay results demonstrated that knockdown of NEAT1 declined the cell apoptosis rate (Figure 2c and d), and the apoptosis-related protein expressions of bax, cleaved caspase-3 and bcl-2 exhibited the consistent trend. Also, the IL-1β, IL-6 and TNF-α mRNA levels were notably inhibited by si-NEAT1 (figure 2f).

### NEAT1 directly targets miR-211-5p

**Figure 3.**
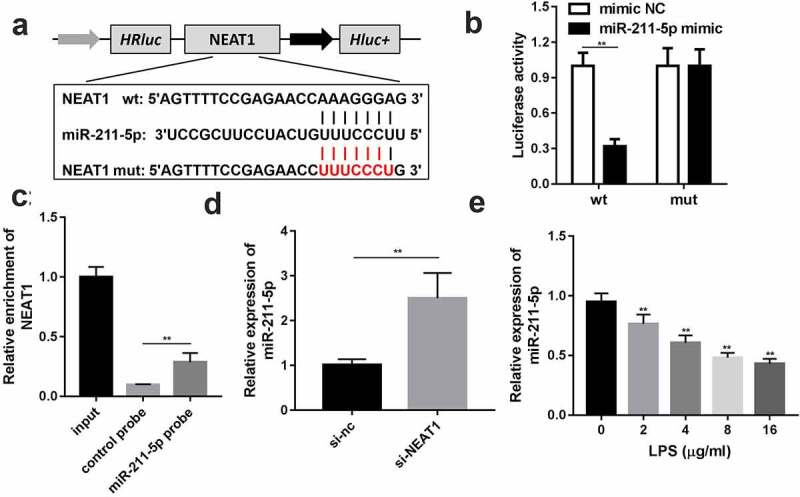
NEAT1 directly targets miR-211-5p. (a) Wild and mutant type of NEAT1 luciferase reporters were constructed and co-transfected with miR-211-5p into the cells. (b) Luciferase activities of the wild and mutant groups. (c) RT-qPCR was used to detect the enrichment of NEAT1 in miR-211-5p biotin-labeled group and control group. (d) MiR-211-5p expression level of the cells transfected with si-NEAT1. (e) MiR-211-5p levels of the cells incubated with different concentrations of LPS were detected. Three independent experiments were carried out. **P < 0.01, vs. mimic NC, control probe, si-nc or 0 μg/ml LPS. LPS, lipopolysaccharide; si, small interference RNA; NEAT1, nuclear enriched abundant transcript 1; wt, wild type; mut, mutant type; nc, negative control

Through bioinformatic approaches, miR-211-5p was predicted to have potential binding sites with NEAT1; wild and mutant types of NEAT1 were designed and synthesized to confirm the prediction (Figure 3a). Luciferase reporter assay results turned out that the miR-211-5p and wild-type NEAT1 co-transfected group exhibited evidently lower luciferase activity than other groups (Figure 3b). The targeted relationship was further validated by RNA pull-down assay, and the biotin-labeled miR-211-5p group enriched a higher level of NEAT1 than the negative control group (Figure 3c). The expression level of miR-211-5p was remarkably lifted via NEAT1 knockdown (Figure 3d) and abated by LPS (Figure 3e)

### Downregulation of miR-211-5p reverses the effects of si-NEAT1

**Figure 4.**
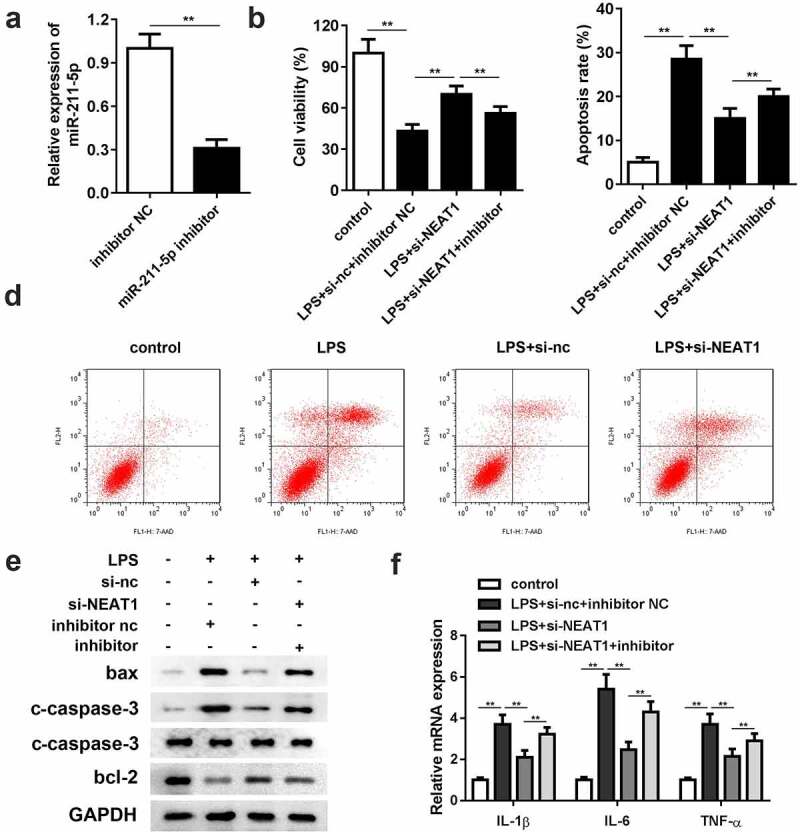
Inhibition of miR-211-5p antagonizes the effects of NEAT1 knockdown on cell viability, apoptosis and inflammation. (a) MiR-211-5p inhibitor was successfully constructed and transfected into the cells. (b) Cell viability and (c and d) apoptosis of the control group and the LPS-evoked cells co-transfected with si-NEAT1 and miR-211-5p inhibitor. (e) The protein expressions of bax, cleaved caspase-3 and bcl-2. (f) mRNA expressions of IL-1β, IL-6, IL-10, and TNF-α in the control group and the LPS-evoked cells co-transfected with si-NEAT1 and miR-211-5p inhibitor. Three independent experiments were carried out. *P < 0.05, vs. control; **P < 0.01, vs. control; ^#^P < 0.05, vs. LPS+si-nc; ^&^P < 0.05, vs. LPS+si-NEAT1+ inh-nc. LPS, lipopolysaccharide; si, small interference RNA; NEAT1, nuclear enriched abundant transcript 1; wt, wild type; mut, mutant type; nc, negative control

Compared with the inhibitor negative control group, the expression level of miR-211-5p was significantly reduced in the miR-211-5p inhibitor group, representing a successful construction of the miR-211-5p inhibitor (Figure 4a). Cell viability was restricted by downregulating miR-211-5p (Figure 4b). With respect to cell apoptosis, miR-211-5p inhibition antagonized the suppressive effect of si-NEAT1 in the LPS-induced cells (Figure 4c and d). The protein expressions of bax and cleaved caspase-3 were promoted, while the protein expression of bcl-2 was reduced in the miR-211-5p inhibitor group. In addition, the inflammation response was substantially exacerbated by miR-211-5p inhibitor (figure 4f).

### MAPK1 is the downstream target gene of miR-211-5p

**Figure 5.**
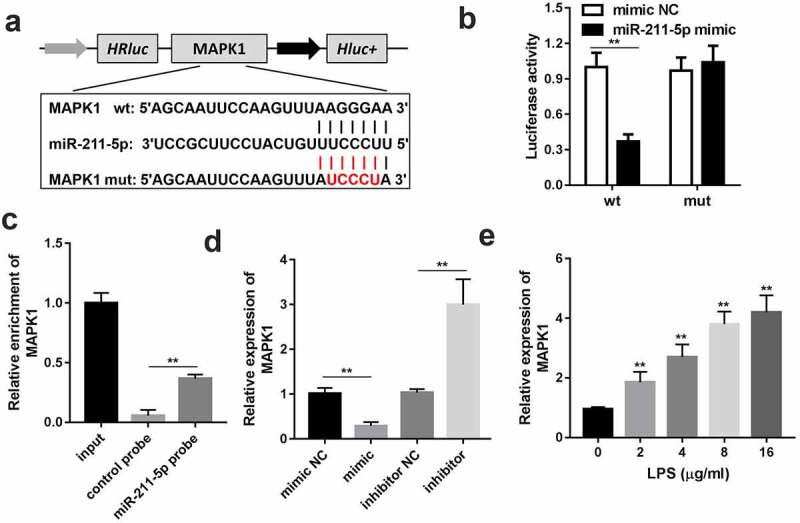
MAPK1 is the downstream target gene of miR-211-5p. (a) Wild and mutant type of MAPK1 luciferase reporters were constructed and co-transfected with miR-211-5p into the cells. (b) Luciferase activities of the wild and mutant groups. (c) RT-qPCR was used to detect the enrichment of MAPK1 in miR-211-5p biotin-labeled group and control group. (d) MRNA expression levels of MAPK1 were analyzed in the miR-211-5p or miR-211-5p inhibitor transfected group. (e) MAPK1 levels of the cells incubated with different concentrations of LPS were detected. Three independent experiments were carried out. **P < 0.01, vs. miR-nc or biotin-nc; ^#^P < 0.05, vs. inhibitor-nc; ^##^P < 0.01, vs. inhibitor-nc. MAPK1, mitogen-activated protein kinase 1; wt, wild type; mut, mutant type; nc, negative control

The wild and mutant type of MAPK1 luciferase reporters were synthesized to confirm the interaction between miR-211-5p and MAPK1 (Figure 5a). The luciferase activity of the wild-type MAPK1 and miR-211-5p co-transfection group was considerably lower than other groups, which verified the authenticity of the interaction (Figure 5b). RNA pull-down assay results further confirmed the interaction (Figure 5c). Subsequently, the expression level of MAPK1 was promoted by LPS (figure 5f).

### Overexpression of MAPK1 antagonizes the effects of NEAT1 knockdown

**Figure 6.**
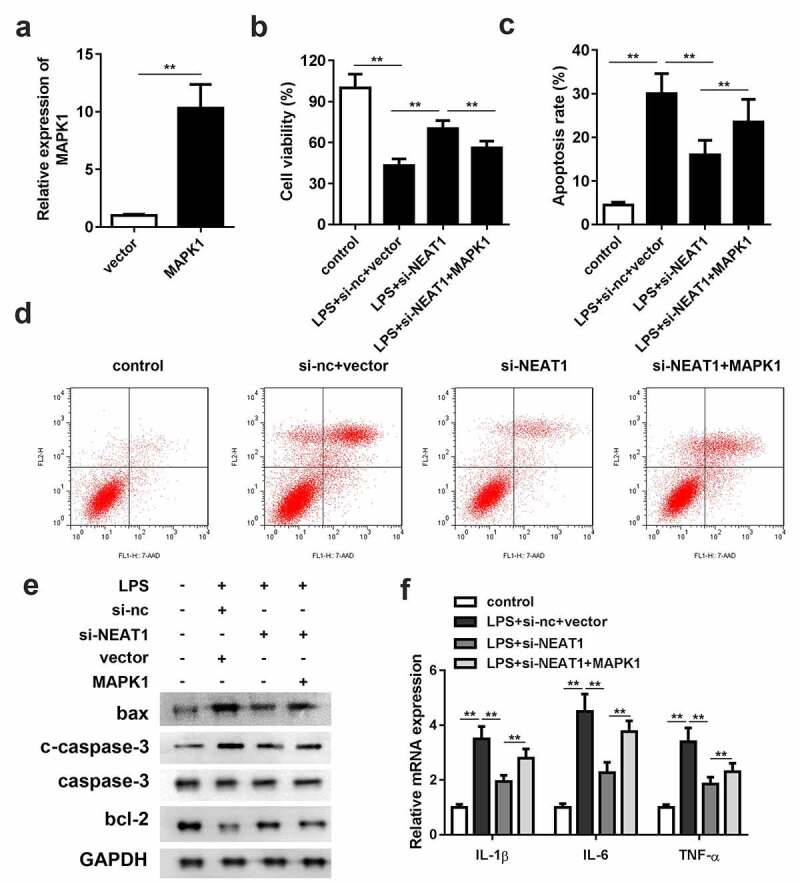
Overexpression of MAPK1 antagonizes the effects of NEAT1 knockdown. (a) MAPK1 overexpression vector was successfully constructed and transfected into the cells. (b) Cell viability and (c and d) apoptosis of the control group and the LPS-evoked cells co-transfected with si-NEAT1 and MAPK1. (e) The protein expressions of bax, cleaved caspase-3 and bcl-2 in the cells co-transfected with si-NEAT1 and MAPK1. (f) mRNA expressions of IL-1β, IL-6, IL-10, and TNF-α in the control group and the LPS-evoked cells co-transfected with si-NEAT1 and MAPK1. Three independent experiments were carried out. *P < 0.05, vs. control; **P < 0.01, vs. control; ^#^P < 0.05, vs. LPS+si-nc; ^&^P < 0.05, vs. LPS+si-NEAT1+ inh-nc. LPS, lipopolysaccharide; si, small interference RNA; NEAT1, nuclear enriched abundant transcript 1; wt, wild type; mut, mutant type; nc, negative control

As shown in Figure 6a, MAPK1 overexpression vector was constructed. Overexpression of MAPK1 partially antagonized the effects of si-NEAT1 on cell viability and apoptosis (Figure 6b-d). The enhancement of bax and cleaved caspase-3 and decrement of bcl-2 protein expressions in the MAPK1 overexpression group suggested that MAPK1 accelerated cell apoptosis. Furthermore, the mRNA expression levels of the inflammasomes were effectively increased by MAPK1 (figure 6f).

## Discussion

Numerous evidences have declared that lncRNA NEAT1 is a significant regulator of inflammasome activated and regulatory processes. For example, NEAT1 was found to exacerbate acute lung injury (ALI) and acute respiratory distress syndrome (ARDS) by inducing alveolar epithelial cell injury and inflammation via HMGB1/RAGE axis. Knockdown of NEAT1 restricted LPS-induced transcripts and production of inflammatory cytokines IL-6, IL-1beta, and TNF-alpha [17]. Neat1 associated with the NLRP3, NLRC4, and AIM2 inflammasomes in macrophages to enhance their assembly and subsequent pro-caspase-1 processing, which suggested Neat1 as a common regulatory factor for inflammasome stimuli [18]. Also, NEAT1 was greatly upregulated in the spinal cord tissues of chronic constriction injury (CCI) rats. Knockdown of NEAT1 could alleviate neuropathic pain behaviors and neuroinflammation via inhibiting IL-6, IL-1beta, and TNF-alpha in CCI rats [19]. In our current research, we found that NEAT1 expression level was related to LPS treatment; knockdown of NEAT1 displayed to attenuate cell apoptosis and inflammation in LPS-evoked cells. Videlicet of our results on the connection between NEAT1 expression and inflammation was in accordant with previous studies.

MiR-211-5p was first identified as a diagnostic biomarker of melanoma [8]. With the deepening of researches on miR-211-5p, other biological functions had been reported. MiR-211-5p was able to inhibit cortical neuron differentiation and survival via NUAK1 repression, which might contribute to the synaptic failure, neuronal loss and cognitive dysfunction in Alzheimer’s disease (AD) [20]. Furthermore, miR-211-5p could serve as a potential therapeutic target via targeting SETBP1 [21] or SIRT1 [22] for breast cancer. Except for tumors, miR-211-5p also contributed to the remission of inflammation. Early type 1 diabetes was characterized by insulitis and pancreatic beta cell destruction. During the progression of insulitis, the inflammatory cytokines (IL-1β, IFN-γ) are released and expression of BCL2 protein was promoted [23]. Overexpression of miR-211-5p was disclosed to inhibit beta cells apoptosis and inflammatory responses in early type 1 diabetes [24]. Lately, Zhang et al. [25] confirmed that miR-211-5p expression level was decreased in SCI rat model, while the upregulation of miR-211-5p relieved neuron apoptosis, inflammation and endoplasmic reticulum stress post SCI in rats. Herein, we validated that NEAT1 served as a sponge for miR-211-5p, and inhibition of miR-211-5p reversed si-NEAT1 effects on cell viability, apoptosis and inflammation of the LPS-evoked cells.

Mitogen-activated protein kinases (MAPKs) contain three types of regulatory pathways in mammals: extracellular signal-regulated kinase pathway (ERK1/2), stress-activated protein kinase pathway (SAPK1/JNK) and p38MAPK pathway [26], of which ERK1/2 pathway was related to cell apoptosis and inflammation [27–29]. MAPK1 was also known as ERK2; LPS induction was demonstrated to activate the MAPK/ERK pathway, further to promote the inflammatory response in human adipocytes [30], and MAPK1 played an initial role in the secondary injury of SCI as well [31]. In the present study, miR-211-5p was illustrated to directly target MAPK1. Besides, overexpression of MAPK1 partially antagonized the effects of NEAT1 knockdown. Finally, the NEAT1/miR-211-5p/MAPK1 pathway was clarified in LPS-evoked SCI cell model. However, there are still some limitations in our research. We included only cell experiments without *in vivo* experiments. The current results still require further validation in clinical studies in future works.

## Conclusion

In conclusion, the expression level of NEAT1 was elevated in the LPS-evoked SCI cell model, and that knockdown of NEAT1 could remarkably promote cell viability and suppress cell apoptosis and inflammatory responses. NEAT1 served as a molecular sponge for miR-211-5p, inhibition of miR-211-5p antagonized the effects of knockdown NEAT1. Furthermore, the downstream target gene of miR-211-5p was elucidated to be MAPK1. The NEAT1/miR-211-5p/MAPK1 pathway is suggested to be a novel therapeutic strategy and complement the pathogenesis of SCI.

## Highlights

1. The present study elucidates the binding interaction between NEAT1 and miR-211-5p.
2. The targeting between miR-211-5p and MAPK1 was firstly demonstrated.
3. The present study firstly elucidates the role of miR-211-5p in LPS induced PC-12 cell injury.
